# Blocking Microglial Proliferation by CSF-1R Inhibitor Does Not Alter the Neuroprotective Effects of Adoptive Regulatory T Cells in 3xTg Alzheimer’s Disease Mice

**DOI:** 10.3390/cimb46040180

**Published:** 2024-03-26

**Authors:** Seon-Young Park, Nari Cha, Soyoung Kim, Songah Chae, Won-jun Lee, Hyunjae Jung, Hyunsu Bae

**Affiliations:** 1 Department of Science in Korean Medicine, College of Korean Medicine, Graduate School, Kyung Hee University, Seoul 02447, Republic of Korea; psys12@naver.com (S.-Y.P.); chanari1205@naver.com (N.C.); samanda0@nate.com (S.K.); 2 Department of Korean Medicine, College of Korean Medicine, Graduate School, Kyung Hee University, Seoul 02447, Republic of Korea; songah3217@gmail.com (S.C.); wonjun0917@naver.com (W.-j.L.); jeongnow@gmail.com (H.J.)

**Keywords:** Alzheimer’s disease, regulatory T cells, GW2580, microglia

## Abstract

Alzheimer’s disease (AD) is a chronic neurodegenerative disease that causes cognitive impairment. Neuroinflammation induced by activated microglia exacerbates AD. Regulatory T cells (Tregs) play roles in limiting neuroinflammation by converting microglial polarization. Therefore, adoptive Treg therapy is considered an attractive option for neurodegenerative disorders. However, the mechanism underlying Treg therapy via microglial modulation is not fully understood. In this study, we sought to determine whether adoptively transferred Tregs were effective when microglia proliferation was inhibited by using GW2580, which is an inhibitor of CSF1R. We found that inhibition of microglial proliferation during Treg transfer did not alter the therapeutic effects of Tregs on cognitive deficits and the accumulation of Aβ and pTAU in 3xTg-AD mice. The expression of pro- and anti-inflammatory markers in the hippocampus of 3xTg mice showed that GW2580 did not affect the inhibition of neuroinflammation by Treg transfer. Additionally, adoptively transferred Tregs were commonly detected in the brain on day 7 after transfer and their levels decreased slowly over 100 days. Our findings suggest that adoptively transferred Tregs can survive longer than 100 days in the brain, suppressing microglial activation and thus alleviating AD pathology. The present study provides valuable evidence to support the prolonged efficacy of adoptive Treg therapy in AD.

## 1. Introduction

Alzheimer’s disease (AD) is a chronic neurodegenerative disease accompanied by cognitive dysfunction. AD is not only the most common form of dementia in the elderly but is also expected to more than triple between 2000 and 2050 [1,2]. One of the contributing factors that exacerbates the pathogenesis of AD is neuroinflammation. Its complex responses cause neuronal dysfunction and cell death [3,4]. Microglia are cell mediators that drive the inflammatory response in the central nervous system (CNS), along with astrocytes. In the AD brain, amyloid-β (Aβ) and tau activate M1 microglia, inducing a pro-inflammatory response, unlike M2 microglia with immunosuppressive function. This activation in chronic neurodegenerative disease and aging, known as ‘microglial priming’, is involved in microglial proliferation and worsens neurological disorders. Hence, microglia are considered potential therapeutic targets for AD [5,6,7,8].

CD4^+^CD25^+^ regulatory T cells (Tregs) are key controllers of immune homeostasis. Tregs protect against brain damage by limiting neuroinflammation, but their suppressive effect is dysregulated in CNS disorders [9,10]. Therefore, adoptive Treg therapy has been attempted in neurodegenerative animal models, including models of AD, Parkinson’s disease (PD), atrophic lateral sclerosis, and trimethyltin (TMT)-induced neurodegeneration [11,12,13,14]. Indeed, Treg therapy has shown neuroprotective effects accompanied by inhibition of M1 microglial activation in these neurodegenerative diseases. In our previous study, we found that Tregs suppress inflammatory microglia through bystander suppression. Tregs not only inhibited microglial-induced neuroinflammation after adoptive transfer, but also suppressed Aβ-induced microglial activation and inflammatory response in vitro [15].

Surprisingly, a single intravenous transfer of Tregs was sufficient to elicit potent neuroprotective effects in 3xTg-AD mice, even two months after the initial injection. Following this result, we sought to determine whether the neuroprotective effects of Treg therapy were affected in the absence of microglia. To temporarily inhibit microglial proliferation, we used GW2580, which is a colony-stimulating factor-1 receptor (CSF-1R) inhibitor. CSF-1 is involved in the proliferation, survival, and differentiation of macrophages and microglia. GW2580 treatment prevents microglial proliferation without inhibiting survival [16,17].

Here, we explored the neuroprotective effects of Tregs on cognitive function and microglial phenotype in AD mice upon GW2580 treatment. We examined the survival of adoptively transferred Tregs in the inguinal lymph nodes, spleen, blood, lung, liver, kidney, and brain. Maintaining Treg presence in the brain for an extended period could provide prolonged therapeutic benefits by continuously modulating inflammatory processes associated with the neurodegenerative disease [18]. Our findings elucidate the neuroprotective effects of Tregs on AD pathology and suggest that these effects are due to the survival of adoptively transferred Tregs for longer than 100 days that maintain microglia in an inactive phenotype.

## 2. Materials and Methods

### 2.1. Animals

Male 3xTg-AD mice harboring APP (KM670/671NL), PS1 (M146V), and tau (P301L) protein transgenes [B6;129-*Psen1^tm1Mpm^*Tg(APPSwe,tauP301L)1Lfa/J]; non-transgenic-control mice [B6129SF2/J]; Thy1.1 mice [B6.PL-Thy1<a>/CyJ]; and C57BL/6 mice were obtained from The Jackson Laboratory (Bar Harbor, ME, USA). Animals were housed under a 12 h light/dark cycle and had free access to food and water. All animal experiments were conducted according to the guidelines for animal care and the guiding principles for experiments using animals and were approved by the University of Kyung Hee Animal Care and Use Committee, [KHUASP(SE)-21-102] and [KHUASP(SE)-20-240]. All animal studies were reviewed and performed in compliance with the ARRIVE guidelines.

### 2.2. Manufacturing and Adoptive Transfer of Tregs

Bone marrow (BM) leukocytes from femurs and tibiae of C57BL/6J mice were resuspended in medium containing 20 ng/mL granulocyte–macrophage colony-stimulating factor (GM-CSF; R&D Systems, Minneapolis, MN, USA). After 7 days, the BM leukocytes were washed with magnetic-activated cell sorting (MACS) buffer (Miltenyi Biotec, Auburn, CA, USA) and CD11c^+^ dendritic cells (DCs) were isolated using CD11c MicroBeads (Miltenyi Biotec, Auburn, CA, USA). The cells were resuspended at a density of 2 × 10^5^/mL and seeded in 96-well U-bottom plates. For antigen presentation, DCs were treated with 0.5 µM fibrilized Aβ for 24 h. CD4^+^ T cells from C57BL/6J mice splenocytes were isolated using CD4 (L3T4) MicroBeads (Miltenyi Biotec), resuspended at a density of 2 × 10^6^/mL, and added to DCs at a ratio of 10:1 (CD4 T: DC) with 0.4 µg/mL bvPLA2 (Sigma Aldrich, St. Louis, MO, USA). After 4 days, CD4^+^CD25^+^ T cells (Tregs) were isolated and stimulated using the Treg Expansion Kit (Miltenyi Biotec) for 2 weeks.

### 2.3. Drug Treatment and Adoptive Transfer

To confirm that administration of GW2580 induced immediate inhibition of the microglial population, 4-month-old 3xTg-AD mice were administered 80 mg/kg CSF-1R inhibitor GW2580 (Sigma Aldrich) by oral gavage for 3 days. Treatment controls were administered vehicle (0.5% hydroxypropyl cellulose, 0.1% Tween-80). On day 4, the mice were euthanized, and the brains were harvested. After microglial enrichment using a Percoll density gradient, cells were analyzed using flow cytometry. To impair microglial proliferation, 4-month-old 3xTg mice were administered 80 mg/kg GW2580 (Sigma Aldrich) daily for 6 days. Between administrations, at three days after the administration of GW2580, ex vivo expanded 2 × 10^5^ Tregs were adoptively transferred via the tail vein. Total mice *n* = 28 (*n* = 6 (WT), *n* = 6 (3xTg-AD), *n* = 6 (Treg), *n* = 5 (GW2580), and *n* = 5 (GW2580 + Treg), respectively).

### 2.4. Flow Cytometry

For flow cytometry, cells were washed with BD FACS Stain buffer (BD Bioscience, San Jose, CA, USA) and stained with fluorescently labeled antibodies for 30 min at 4 °C in the dark. The following antibodies were used: FITC-Thy1.1 (Invitrogen, Carlsbad, CA, USA), 7AAD (eBioscience, San Diego, CA, USA), and PE-cy7-CD4 (Invitrogen) for Treg trafficking; PE-CD11c (eBioscience) for DCs; PE-Cy5-CD4 (Invitrogen) for CD4 T cells; PE-cy7-CD4 (Invitrogen) and APC-cy7-CD25 (BD Pharmingen, Franklin Lakes, NJ, USA) for Tregs; and 7AAD (eBioscience), PE-cy7-CD11b (BioLegend, San Diego, CA, USA), and BV421-CD45 (BioLegend) for microglia. The samples were washed and analyzed using flow cytometry. The data were acquired using a BD FACSlyric™ (BD Biosciences, San Jose, CA, USA) flow cytometry system and analyzed using the BD FACSuite software 1.2.1 (BD Biosciences).

### 2.5. Passive Avoidance Test (PAT)

Learning and memory were assessed using the PAT. The shuttle box (cat# JD-SI-10) and electric shock generator were purchased from Jeungdo Bio & Plant Co., Ltd. (Seoul, Republic of Korea). The apparatus consisted of a lighted and a dark chamber (20 cm × 20 cm × 30 cm) divided by a door. A lamp (50 W) was placed in one chamber for illumination. Each test involved three separate trials: two training trials and a test trial. On the training day, the mice were placed in the lighted chamber facing away from the door. Upon entering the dark chamber, the door was closed and a foot shock (0.35 mA, 2 s) was delivered to the chamber floor. Thirty seconds after the shock, the mouse was removed from the dark chamber. The latency times once the mice had entered the dark compartment were measured using a stopwatch. The latency time before re-entering the dark chamber was measured up to 300 s. The mice were subjected to trials for two days. On the test day, the mice were placed in the lighted chamber, and the latency time before entering the dark chamber was recorded.

### 2.6. Immunohistochemistry Analysis

For immunohistochemistry, mice were anesthetized with isoflurane (Forane solution; ChoongWae Pharma, Seoul, Republic of Korea) and were transcardially perfused with phosphate-buffered saline (PBS). The brains were postfixed in 4% paraformaldehyde at 4 °C overnight, transferred to 30% sucrose solution, and frozen-sectioned on a sliding microtome into 30 μm thick coronal sections using a cryomicrotome (HM525 NX; Thermo Fisher Scientific, Inc., Waltham, MA, USA). The brain sections were incubated with 99% formic acid for 5 min at room temperature (RT) and then heated in 10 mM sodium citrate buffer (pH 6.0) at 60 °C. After washing with PBS, nonspecific binding was reduced by blocking the sections with 5% bovine serum albumin in Tris-buffered saline with 0.05% Triton X-100 (TBSTr) for 30 min.

To detect microglia, primary antibodies were directed against CD11b (1:500; Santa Cruz Biotechnology, Dallas, TX, USA). The sections were washed with PBS, incubated with the appropriate biotinylated secondary antibody, and processed using an avidin–biotin complex kit (Vectastain ABC kit; Vector Laboratories, Burlingame, CA, USA) for 1 h at RT. The reaction product was visualized with 0.05% diaminobenzidine-HCl (DAB) and 0.003% hydrogen peroxide in 0.1 M phosphate buffer. The labeled tissue sections were subsequently mounted and analyzed under a bright-field microscope (Nikon, Tokyo, Japan).

For immunofluorescence, the sections were incubated with mouse monoclonal 4G8 antibody (1:500; BioLegend), phospho-Tau (1:1000; Invitrogen), Iba1 (1:1000; Abcam, Cambridge, UK), and NOS2 (1:500; Santa Cruz Biotechnology) for 3 days at 4 °C. Brain sections were washed with TBSTr and subsequently incubated for 2 h at RT with Alexa 488- or 594-conjugated IgG secondary antibodies. The slides were mounted with DAPI mounting medium (Vector Laboratories, Burlingame, CA, USA) and examined using an LSM 800 confocal laser-scanning microscope (Carl Zeiss, Oberkochen, Germany). The staining intensity was quantified by measuring the integral density of the region of interest from monochromatic images using ImageJ software 1.52a. The percentage of staining intensity was calculated relative to Tg and multiplied by 100.

### 2.7. RNA Extraction and RT-PCR Assays

Total RNA was isolated from the whole-cerebrum tissues using the easy-BLUE Total RNA extraction kit (iNtRON Biotechnology, Seongnam, Republic of Korea), and cDNA was synthesized using CycleScript reverse transcriptase (Bioneer, Daejeon, Republic of Korea). The samples were prepared for real-time RT-PCR using the SensiFAST SYBR no-ROX kit (Bioline, Daejeon, Republic of Korea). Real-time quantitative PCR was performed using CFX Connect System (Bio-Rad, Hercules, CA, USA). The cycling conditions were 1 cycle at 95 °C for 30 s, 49 cycles at 95 °C for 10 s, and 55 °C for 30 s, followed by a melting curve at 95 °C for 10 s and 50 °C for 5 s, and then a gradual increase until 95 °C was reached. The expression levels of the target mRNAs were normalized to the expression levels of mouse β-actin, a housekeeping gene used as an endogenous control. All fold-changes were expressed relative to the wild-type group.

The primer base sequences are listed in Table 1.

### 2.8. Thy 1.1 Treg Trafficking

For Treg trafficking, Tregs were isolated from splenocytes of Thy1.1 mice using MACS according to the manufacturer’s protocols (CD4^+^CD25^+^ Regulatory T Cell Isolation Kit; Miltenyi Biotec). Five sixteen-week-old 3xTg mice (Thy 1.2^+^) received adoptive transfer of 1 × 10^6^ Thy 1.1^+^ Tregs. Mice were sacrificed at 3, 7, 14, 28, 56, 84, and 112 days after receiving adoptive transfer of Thy 1.1^+^ Treg, and the inguinal lymph nodes, spleen, blood, lung, kidney, liver, and brain were harvested. T cells were enriched using 30–70% Percoll (Cytiva, Marlborough, MA, USA) density gradient centrifugation and Debris Removal Solution (Miltenyi Biotec) from the lung, kidney, liver, and brain. Adoptively transferred Tregs were analyzed using flow cytometry.

### 2.9. Statistical Analysis

GraphPad Prism 5.01 software (GraphPad Software Inc., San Diego, CA, USA) was used for statistical analyses. One-way analysis of variance (ANOVA) followed by Tukey’s multiple comparison tests were performed. A two-tailed Student’s unpaired test was performed for comparing the two groups. Multiple comparisons within groups were analyzed using a two-way ANOVA, followed by Bonferroni post hoc tests. All experiments were performed in a blinded manner and were repeated independently under identical conditions. Statistical significance was set at *p* < 0.05.

## 3. Results

### 3.1. CSF-1R Inhibition Reduced Microglia and Macrophage Population in 4-Month 3xTg-AD Mice

**Figure 1:**
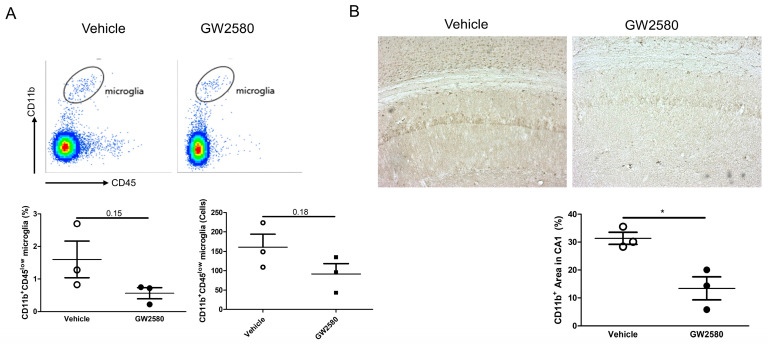
Administration of GW2580 reduces microglial population. (**A**) Four-month-old 3xTg mice were administered GW2580 by oral gavage for 3 days. On day 4, CD11b^+^CD45^low^ microglia were detected in the whole cerebrum. (**B**) The area of CD11b^+^ microglia in the hippocampal CA1 region was reduced after GW2580 administration. Magnification = 10X. Data are presented as the mean ± SEM. Statistical analyses were conducted using an unpaired *t*-test; * *p* < 0.05. *n* = 3.

To determine whether short-term administration of GW2580 induced immediate inhibition of the microglial population, 4-month-old 3xTg-AD mice were administered GW2580 for 3 days. On day 4, the population of microglia in the whole cerebrum was detected using flow cytometry (Figure 1A). Short-term administration of GW2580 slightly decreased the population of CD11b^+^CD45^low^ microglia but the result was not statistically significant. We observed CD11b^+^ microglia in the hippocampus (Figure 1B). The area of CD11b^+^ significantly decreased after GW2580 administration.

### 3.2. Adoptive Transfer of Tregs Attenuates Learning and Memory Impairment in GW2580-Treated 3xTg-AD Mice

**Figure 2:**
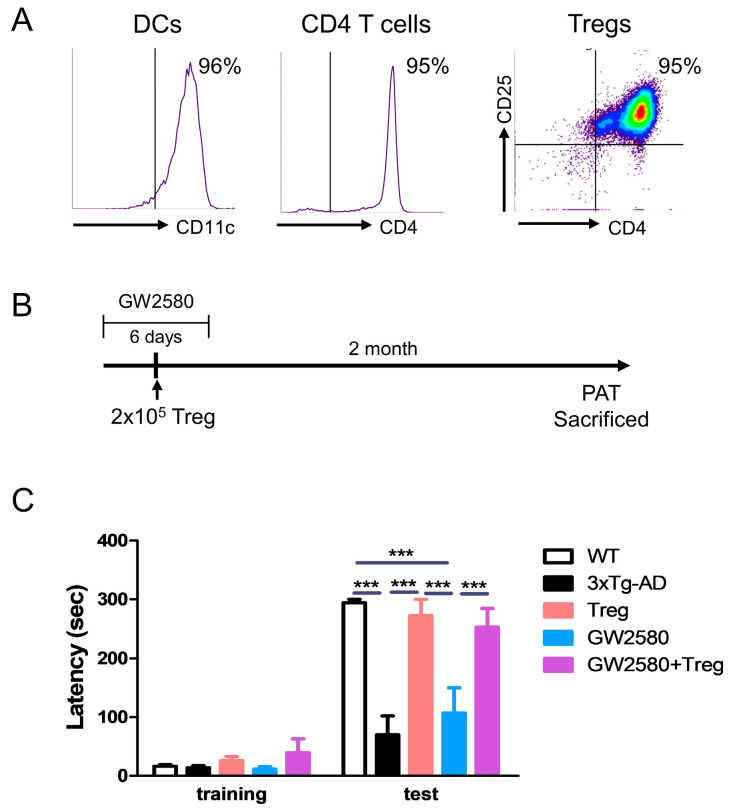
Regulatory T cells (Tregs) modulate behavior deficits in GW2580-treated AD mice. (**A**) CD11c^+^ dendritic cells (DCs) were isolated from mouse bone marrow and treated with Aβ. Aβ-specific DCs were co-cultured with CD4^+^ cells isolated from the spleen (1:10). After 4 days, CD4^+^CD25^+^ regulatory T cells were isolated. (**B**) Ex vivo expanded Tregs were adoptively transferred to GW2580-treated 3xTg-AD mice. (**C**) After 2 months, a passive avoidance test was performed and the latency (s) was measured. Data are presented as the mean ± SEM. Statistical analyses were conducted using two-way ANOVA; *** *p* < 0.001. *n* = 4–6.

Aβ-specific Tregs were produced as previously described [14]. The purity of the isolated cells was analyzed using flow cytometry (Figure 2A). To inhibit microglial proliferation during Treg transfer, the CSF-1R inhibitor GW2580 was administered to 3xTg-AD mice for 6 days before and after Treg transfer. After two months of Treg transfer, PAT was performed (Figure 2B). The adoptive transfer of Tregs significantly improved the learning and memory impairment in 3xTg-AD mice (Figure 2C). GW2580 treatment did not improve learning and memory impairment, but adoptive transfer of Tregs was effective in GW2580-treated 3xTg-AD mice.

### 3.3. Adoptive Transfer of Tregs Attenuates AD Pathology in GW2580-Treated 3xTg-AD Mice

**Figure 3:**
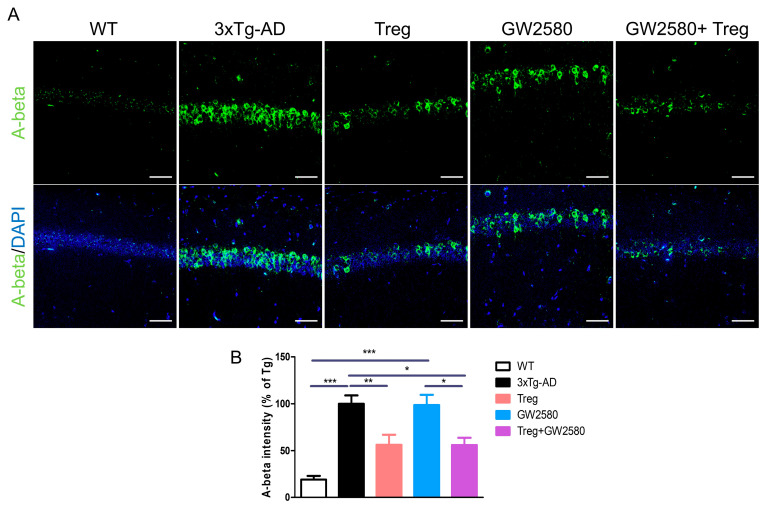
Tregs modulate Aβ accumulation in GW2580-treated AD mice. To inhibit microglial proliferation during Treg transfer, the CSF-1R inhibitor GW2580 was administered to 3xTg-AD mice for 6 days. Between administrations, at three days after the administration of GW2580, ex vivo expanded 2 × 10^5^ Tregs were adoptively transferred via the tail vein. After 2 months, mice were anesthetized with isoflurane and were transcardially perfused with PBS, and the brains were harvested and postfixed for immunohistochemistry analysis. (**A**) Aβ accumulation was observed in the hippocampus. (**B**) The intensity of Aβ was measured. Data are presented as the mean ± SEM. Statistical analyses were conducted using one-way ANOVA; * *p* <0.05, ** *p* <0.01, and *** *p* < 0.001. *n* = 4–6. Scale bar: 50 μm.

Abnormal accumulation of Aβ in brain areas, especially in the hippocampus, is a major feature of AD [19]. Therefore, we examined Aβ peptides in the hippocampus (Figure 3A). As expected, Aβ accumulation detected in 3xTg-AD mice was significantly decreased by the adoptive transfer of Tregs, regardless of GW2580 treatment (Figure 3B).

**Figure 4:**
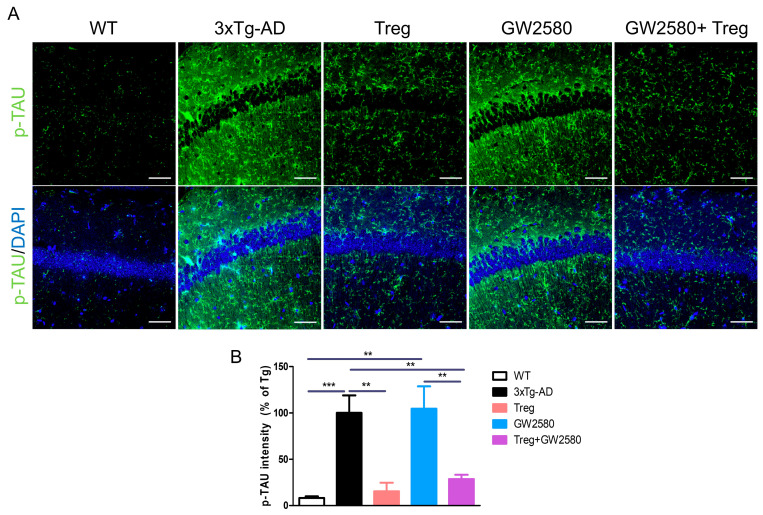
Tregs modulate phosphorylated tau (pTAU) accumulation in GW2580-treated AD mice. (**A**) To detect AD pathology, brain sections were labeled for pTAU. (**B**) The intensity of pTAU in the hippocampus was measured. Data are presented as the mean ± SEM. Statistical analyses were conducted using one-way ANOVA; ** *p* <0.01 and *** *p* < 0.001. *n* = 4–6. Scale bar: 50 μm.

Similarly, the expression of phosphorylated tau was assessed in the hippocampus (Figure 4A). Treg transfer significantly reduced the hyper-phosphorylation of tau, regardless of GW2580 treatment (Figure 4B). These results support the contention that adoptively transferred Tregs attenuate AD pathology, even when microglial proliferation is suppressed.

### 3.4. Adoptive Transfer of Tregs Modulates Microglial Polarization in GW2580-Treated 3xTg-AD Mice

**Figure 5:**
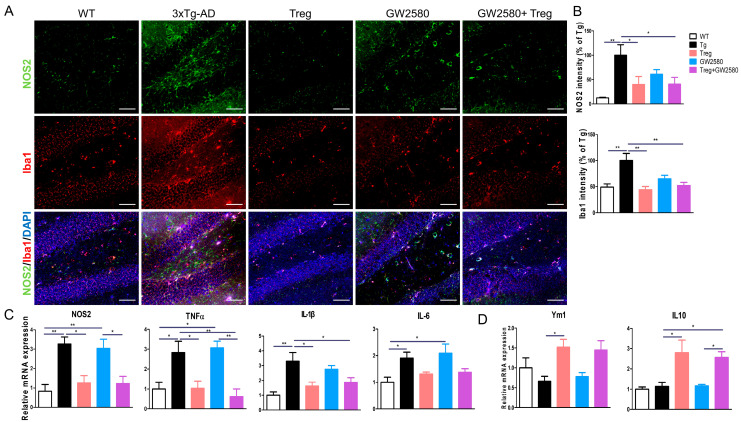
Tregs reduce pro-inflammatory microglia in GW2580-treated AD mice. (**A**) The expression of NOS2 and Iba1 was detected in the hippocampal dentate gyrus (DG). (**B**) NOS2 and Iba1 intensities were measured. (**C**,**D**) The relative mRNA expression of pro-inflammatory markers (NOS2, TNFα, IL-1β, and IL-6) was analyzed in the whole cerebrum. Data are presented as the mean ± SEM. Statistical analyses were conducted using one-way ANOVA; * *p* < 0.05 and ** *p* < 0.01. *n* = 4–6. Scale bar: 50 μm.

To assess the activation of pro-inflammatory M1 microglia, brain sections were immunostained for NOS2 and Iba1 (Figure 5A). While the intensities of NOS2 and Iba1 significantly increased in 3xTg-AD mice, as expected, lower intensities were observed in GW2580-treated mice. Adoptive transfer of Tregs significantly reduced NOS2 and Iba1 intensities, regardless of GW2580 treatment (Figure 5B). At the gene expression level, we found that highly expressed M1 markers, including NOS2, TNFα, and IL-1β, in 3xTg-AD mice were downregulated both in Treg- and GW2580-treated Treg mice (Figure 5C). In contrast, the expression of M2 markers, such as Ym1 and IL-10, was upregulated by Treg transfer, but, similarly, there were no effects after GW2580 treatment (Figure 5D).

### 3.5. Adoptively Transferred Tregs Survive up to 112 Days in Several Tissues of 3xTg-AD Mice

**Figure 6:**
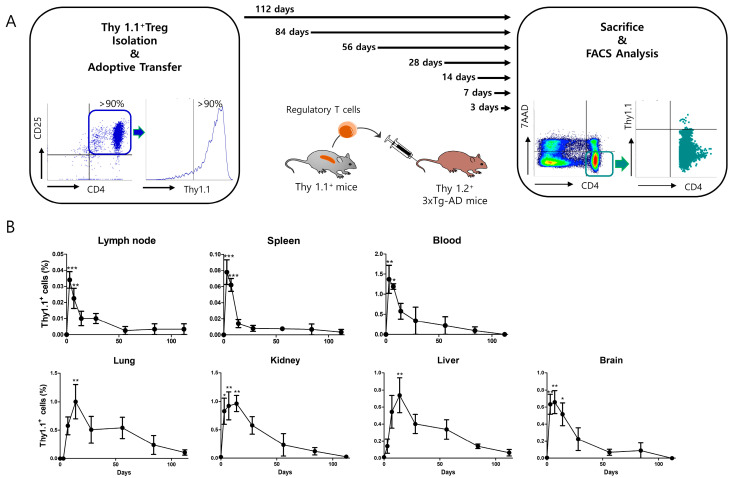
Tissue distribution and survival of adoptively transferred Tregs. (**A**) Thy1.1^+^ Tregs were isolated from the splenocytes of Thy1.1 mice and adoptively transferred to five Thy1.2^+^ 3xTg-AD mice. Mice were sacrificed at 3, 7, 14, 28, 56, 84, and 112 days after receiving adoptive transfer of Thy1.1^+^ Treg. 7AAD-CD4+Thy1.1^+^ cells were then detected in the inguinal lymph nodes, spleen, blood, lung, liver, kidney, and brain from each mouse using flow cytometry. (**B**) The proportion of adoptively transferred Thy1.1^+^ Tregs over time in each tissue was analyzed. Data are presented as the mean ± SEM. Statistical analyses were conducted using one-way ANOVA; * *p* < 0.05, ** *p* < 0.01. *** *p* < 0.001. *n* = 3–5.

To determine the survival of adoptively transferred Tregs in various mouse tissues, Thy1.1^+^ Tregs were adoptively transferred to 3xTg-AD mice (Figure 6A). After 3–112 days of transfer, mice were sacrificed, and transferred Tregs were detected using flow cytometry. In the lymph nodes, spleen, and blood, transferred Tregs were detected the most on day 3, and their levels decreased rapidly over time. In the lung, kidney, liver, and brain, transferred Tregs were most frequently detected on day 7; their levels decreased relatively slowly, and were hardly detected on day 112. These results suggest that adoptively transferred Tregs were present in the brains of 3xTg-AD mice for more than one month (Figure 6B).

## 4. Discussion

Regulatory T cells (Tregs), which are a small subset of T cells, can regulate the immune system, as well as maintain immune homeostasis [15]. Numerous studies have demonstrated the potential of Treg therapy in various diseases, including graft-versus-host disease, colitis, type 1 diabetes, and multiple sclerosis [20,21,22,23]. Recently, Yeapuri et al. reported that the adoptive transfer of amyloid-β (Aβ)-specific Tregs attenuates Alzheimer’s disease (AD) in APP/PS1 mice expressing an amyloid precursor protein and a presenilin 1 [24]. Furthermore, when Tregs were intravenously administered to ischemic mice, the brain hemorrhagic transformation was alleviated, suggesting a neuroprotective effect mediated by the adoptive transfer of Tregs [25]. Nevertheless, several challenges remain, such as the suppressive mechanism of adoptively transferred Tregs [26,27]. In previous studies, we revealed the therapeutic effects of Tregs were accompanied by microglial modulation in AD- and TMT-induced neuroinflammation models [11,14]. In the follow-up study, we sought to explore the interaction between adoptively transferred Tregs and microglia. Thus, to investigate when adoptively transferred Tregs affect microglial polarization, we trafficked adoptively transferred Tregs and inhibited the proliferation of microglia that act as mediators of Treg effects. We reached the following conclusions. First, adoptively transferred Tregs are most frequently observed in the brain 3–7 days after transfer, especially at 7 days after transfer. Second, suppression of microglial proliferation during Treg transfer does not affect the efficacy of Treg therapy. Therefore, our findings support the contention that adoptively transferred Tregs induce microglial modulation after 7 days of transfer and then exert neuroprotective effects in AD.

In our recently published data, we attempted the depletion of adoptively transferred Tregs using the diphtheria toxin in DEpletion of REGulatory T cells (DEREG) mice, and found that adoptively transferred Tregs must survive for more than 7 days to induce neuroprotective effects [15]. In the current study, we tracked adoptively transferred Tregs in several tissues. Our data show that adoptively transferred Tregs are the most present in the brain on day 7; their levels then gradually decreased, but Tregs survived even 100 days after transfer. The effects of adoptive Tregs on neurodegenerative diseases have been demonstrated over the years. However, the current challenge in Treg therapy is to maximize its effectiveness and efficiency [28,29,30]. We thus prepared antigen-specific Tregs using a disease-specific antigen, and confirmed that expanded antigen-specific Tregs have therapeutic effects in TMT-induced hippocampal neurodegeneration [14]. In this study, we prepared antigen-specific Tregs using fibrilized Aβ, which is an AD-specific antigen found in the brain of patients with AD. After expansion, Aβ-specific Tregs were adoptively transferred to 3xTg-AD mice.

As demonstrated in human AD patients, microglial proliferation increases in 3xTg-AD mice [31,32,33]. Therefore, CSF-1R inhibitors that suppress microglial proliferation, such as GW2580 and PLX3397, have been explored as therapeutic agents for the treatment of AD. Because the effect of these drugs is temporary, prolonged administration is required; for example, GW2580 for 3 months and PLX3397 for 28 days [31,34]. Since the purpose of this study was to confirm the efficacy of Tregs in conditions with suppressed microglial proliferation, animal treatment was limited to 6 days to exclude the effects on AD pathology by GW2580 itself. Microglial repopulation started 3 days after CSF-1R inhibitor withdrawal [35]. This means that microglia repopulate 7 days after Treg transfer. Therefore, our results imply that Tregs should begin to take effect on the microglia 7 days after adoptive transfer. To determine the time period at which Tregs modulate microglia, further studies will be required with GW2580 treatments for several periods after Treg injection.

Accumulation of Aβ is a typical hallmark of AD and is evaluated in AD trials with memory impairment. Hyper-phosphorylated and aggregated tau is also a neuropathological feature of AD that correlates with neurodegeneration and cognitive deficits [36]. Therefore, we performed PAT and immunostaining of Aβ and phosphorylated tau to measure AD pathology. As expected, Aβ-specific Tregs showed neuroprotective effects against memory deficits and Aβ accumulation.

Microglia are immune cells that reside in the brain and are known to be activated in AD. During AD progression, microglia undergo conversion to the M1 rather than the M2 phenotype and contribute to the progression of the disease. Activated M1 microglia may be detrimental to AD by inducing the expression of pro-inflammatory cytokines, such as IL-1β and TNFα [37,38]. It has been demonstrated that Tregs ameliorate brain damage by alleviating microglial inflammation in neuromyelitis optica spectrum disorder and intracerebral hemorrhage model mice [39,40]. Additionally, a study has shown that expanded human Tregs induce monocyte activation into the anti-inflammatory M2 phenotype [41]. Microglial inflammation is an important indicator of AD and a target for Treg function. In line with other results, Aβ-specific Tregs effectively restored the M1/M2 balance, regardless of GW2580 treatment. Notably, different to the results related to memory impairment and Aβ accumulation, the fluorescence intensity of NOS2 and Iba1 in the hippocampus slightly decreased after treatment with GW2580 alone. However, the reduction was not significant and was not reproduced using mRNA expression. We assume that short-term inhibition by GW2580 in conditions of microglial priming had a slight effect after two months.

## 5. Conclusions

We found that the survival time of adoptively transferred Tregs to 3xTg-AD mice is sufficient to keep microglia inactive in the brain, resulting in the suppression of AD pathology. These results provide valuable evidence to support the prolonged efficacy of adoptive Treg therapy in AD.

## Figures and Tables

**Table 1:** Primer base sequences for RT-PCR.

Gene Name	Forward Primer Sequence (5′–3′)	Reverse Primer Sequence (5′–3′)
*β-actin*	GTG CTA TGT TGC TCT AGA CTT CG	ATG CCA CAG GAT TCC ATA CC
*NOS2*	AGG ACA TCC TGC GGC AGC	GCT TTA ACC CCT CCT GTA
*TNF-α*	GGC AGG TTC TGT CCC TTT CAC	TTC TGT GCT CAT GGT GTC TTT TCT
*IL-1β*	AAG CCT CGT GCT GTC GGA CC	TGA GGC CCA AGG CCA CAG G
*IL-6*	TTC CAT CCA GTT GCC TTC TTG	GGG AGT GGT ATC CTC TGT GAA GTC
*Ym1*	TGG AGG ATG GAA GTT TGG AC	GAG TAG CAG CCT TGG AAT GT
*IL-10*	CAG CCG GGA AGA CAA TAA CTG	CCG CAG CTC TAG GAG CAT GT

## Data Availability

All data generated or analyzed during this study are included in this published article.

## Abbreviations

AD: Alzheimer’s disease; one-way ANOVA: one-way analysis of variance; Aβ: amyloid-β; CNS: central nervous system; CSF-1R: colony-stimulating factor-1 receptor; DAB: diaminobenzidine; MACS: magnetic-activated cell sorting; PAT: passive avoidance test; PBS: phosphate-buffered saline; PD: Parkinson’s disease; pTAU: phosphorylated tau; RT: room temperature; Tregs: regulatory T cells; TMT: trimethyltin; TBSTr: Tris-buffered saline with 0.05% Triton X-100.
